# Ecology of Tularemia in Central US Endemic Region

**DOI:** 10.1007/s40475-016-0075-1

**Published:** 2016-06-16

**Authors:** Rinosh J. Mani, Rebecca J. Morton, Kenneth D. Clinkenbeard

**Affiliations:** 1 Diagnostic Center for Population and Animal Health, Michigan State University, Lansing, MI USA; 2 Department of Veterinary Pathobiology, Center for Veterinary Health Sciences, Oklahoma State University, Stillwater, OK USA

**Keywords:** Tularemia, Ecology, Central USA, Feline tularemia, Rabbit-tick cycle

## Abstract

Tularemia is a zoonotic disease that occurs in the Northern Hemisphere caused by the gammabacterium *Francisella tularensis*. The most severe form of human tularemia occurs in the central USA and involves a rabbit enzootic cycle, ixodid tick vectors, and *F. tularensis* subspecies *tularensis* genotype A1. Enzootic tularemia is thought to have a spring-summer seasonality corresponding to the questing activity of its primary tick vectors. Domestic cats, another common incidental host, acquire the infection by preying on infected rabbits. The seasonality of tularemia in cats, which demonstrate a bimodal seasonal incidence curve with peaks in the spring and late summer-fall, may serve as a surrogate for the seasonality of the disease in its enzootic host. Human tularemia shows a unimodal late spring, early summer peak, which correlates to the seasonal questing activity of tick vectors of human tularemia. This difference in seasonality suggests that different tick species or tick life stages are involved in maintenance of the enzootic rabbit-tick cycle.

## Introduction

Tularemia is a zoonotic disease that occurs in the Northern Hemisphere caused by the gram-negative bacterium *Francisella tularensis* [1–3]. It was first reported as a human pathogen in the USA in the early twentieth century [4]. Subsequently, the agent has been isolated from more than 300 different species of mammals, birds, reptiles, amphibians, fish, and invertebrates [5]. Because of its low infectious dose, ability to infect via aerosol, and history of being developed as a bioweapon, *F. tularensis* has been designated as Tier 1 Select Agent by the Centers for Disease Control and Prevention (CDC, Atlanta, Georgia, USA) [6].

In North America, tularemia is caused by infection with either *F. tularensis* subspecies *tularensis* (type A) or *F. tularensis* subspecies *holarctica* (type B); however, in Eurasia where type A is not observed, the primary subspecies causing tularemia is type B, with fewer cases due to *F. tularensis* subspecies *mediasiatica*. Types A and B are both found in the central USA but differ in their virulence and ecological niche. Type A strains have been characterized further into genotypes that differ in virulence, ecological niche, and disease transmission patterns [7, 8]. Type A1is found in the central states endemic region and is the most virulent and invasive subtype, while type A2 is observed in the western USA and is less virulent. Type B is associated with water bodies especially the upper Mississippi and Missouri Rivers, high rainfall areas, rodents and ticks, whereas type A1 is associated with lagomorphs, cats, and ticks [7, 8, 9•]. This review will focus on the ecology and transmission of *F. tularensis* subspecies *tularensis* type A1 in central USA comprising principally Arkansas, Kansas, Missouri, and Oklahoma. A comprehensive review of the ecology of *F. tularensis* in North America is beyond the scope of this review, and readers are advised to the following excellent reviews on the topic [1, 10–14].

The ecology of human tularemia in the USA has changed over the past century. During the first half of the twentieth century, the majority of human cases were attributed to exposure to infected lagomorphs, while during the latter part of the century, human tularemia has been predominantly a tick-vectored disease [1]. Overall, the incidence of human tularemia in the USA has decreased over these years, while the number of human cases in the central USA has remained steady [1, 6]. The central US tularemia endemic region accounts for >60 % of the total human tularemia cases in the USA, and since the 1980s, most human cases in this region have been attributable to tick bites [1, 6, 15].

The majority of human infections in the central endemic region occurs during the summer months and correlates with peak questing activity of the predominant tick species in this region that feed on humans, namely *Dermacentor variabilis* and *Amblyomma americanum* [1, 16, 17]. Both of these tick species have been shown to be experimental vectors, have been found to be infected with *F. tularensis* in nature, and may serve as inter-seasonal reservoirs [18, 19•, 20–24]. Human tularemia is a reportable disease for the Centers for Disease Control and Prevention resulting in epidemiologic records being available to reconstruct seasonal incidence patterns of tularemia as a surrogate for endemic cycle in rabbits where there is limited direct data available for assessment. Another source of data for seasonal incidence of rabbit tularemia is incidental infection in domestic cats, which acquire tularemia directly through predation of infected rabbits [25–29].

Rabbits, particularly eastern cottontails, have been historically accepted as the reservoir of *F. tularensis*; however, the high mortality rates when exposed to type A1 and the transient bacteremia in infected rabbits question their reservoir status [30•]. *A. americanum* and *D. variabilis* have been shown to carry the bacteria transstadially in high numbers for extended periods of time, and ixodid ticks have a long coevolution association with *Francisellaceae* as demonstrated by *Francisella*-like endosymbionts in a variety of tick species [18, 19•, 24, 31, 32]. However, ticks might not be a complete reservoir as evident from the reports of negative fitness costs associated with virulent strains of *F. tularensis* and the lack of transovarial transmission of *F. tularensis* in the implicated tick vectors [18, 19•, 20, 33]. It is logical to assume that other unknown reservoir hosts or climatic factors that are yet to be determined would play a role in the ecology of tularemia.

## Feline Tularemia

Of domestic animals, the cat appears to be particularly susceptible to infection by *F. tularensis,* which occurs by ingestion of infected animals, the rabbit being by far the most common source. This high susceptibility of cats to tularemia is supported by numerous case reports in the literature for feline tularemia compared to those for dogs, assuming that dogs inhabiting the same environments would have a similar prevalence if their susceptibility was similar [28, 34, 35]. In addition, exposure to infected cats, but not dogs, is a primary source of non-vector transmitted human tularemia [3, 25, 26, 29, 36–39]. Based on the observation that most cases of feline tularemia are oropharyngeal and that predation of rabbits is the source for these infections, the incidence and seasonality of feline tularemia may serve as a surrogate for better understanding the ecology of the endemic rabbit tularemia cycle, where direct records are lacking and difficult to obtain [27, 28, 35, 40].

**Fig. 1:**
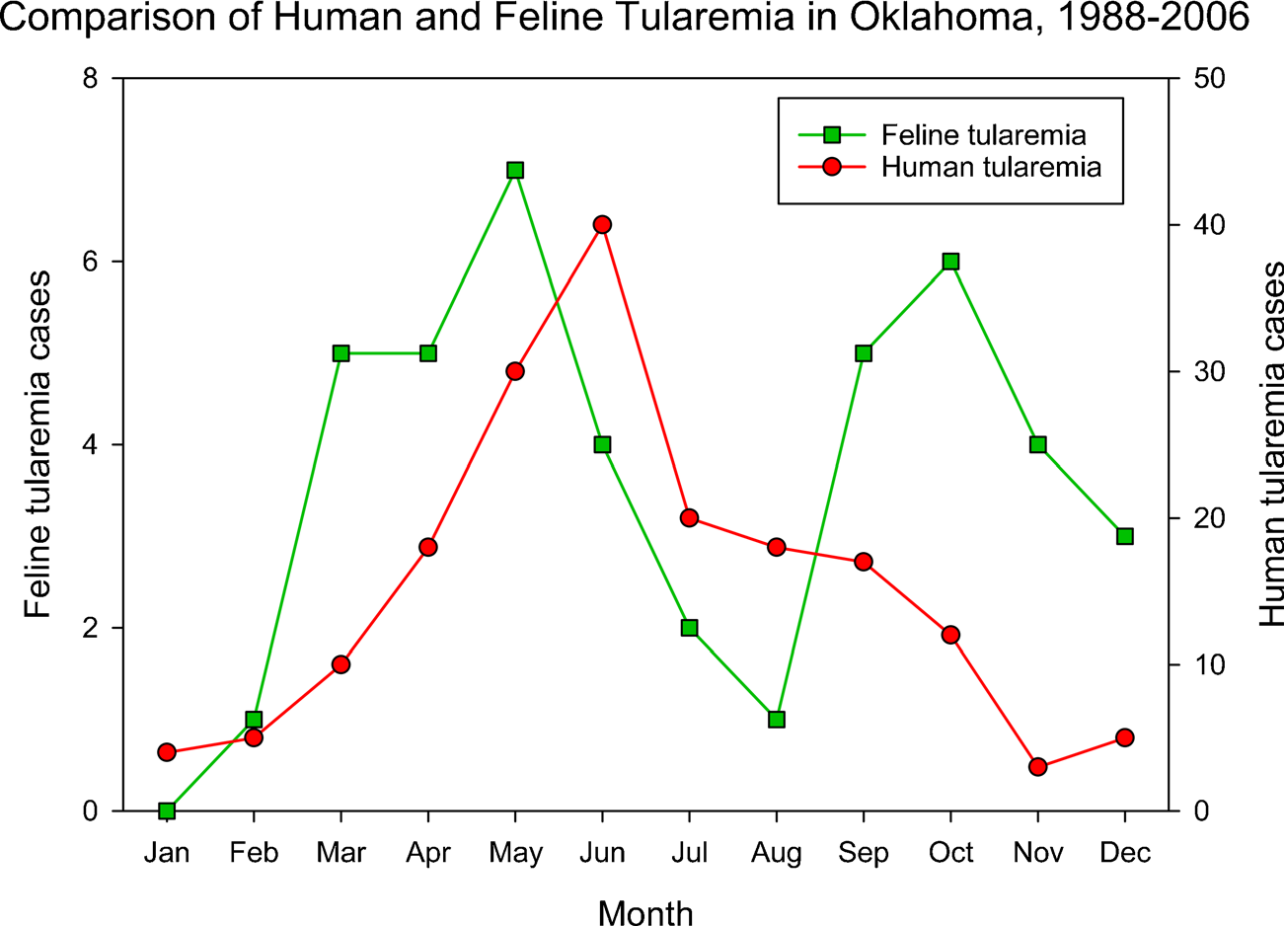
Comparison of human and feline tularemia in Oklahoma, 1988–2006

As shown in Fig. 1, feline tularemia has March to June and September to November seasonal incidence peaks with August and January nadirs. The data for feline tularemia is for clinical cases with microbial culture or IFAT testing of fresh or fixed tissue collected by one of us (RJM). The actual prevalence of feline tularemia is likely markedly underestimated by this data, as feline tularemia is not a reportable disease and because cats in rural areas are far more likely to be infected, but owners are less likely to consult a veterinarian for treatment and diagnosis [35]. Another factor in underestimation is the acuteness and high mortality of the disease in cats.

The seasonal incidence peaks for feline tularemia may reflect the disease incidence in the definitive rabbit hosts for the central US tularemia endemic region. The incubation period for oral ingestion of infected hosts for cats is in the range of 1 to 7 days, suggesting that the peak seasonality for tularemia in rabbits occurs in May and October. The late spring and early summer peak in tularemia incidence has been appreciated from the similar seasonal incidence peak for human tularemia (data provided by Dr. Kristi Bradley, Oklahoma Department of Health, personal communication). This peak has been attributed to the seasonal questing activity of the tick vectors thought to be the primary vectors for maintenance of enzootic tularemia in the central USA, namely *D. variabilis* and *A. americanum*.

The late summer and fall incidence peak of feline tularemia is more difficult to correlate with our current understanding of enzootic tularemia. This peak of tularemia is, like the spring and early summer peak, associated with feline predation of infected rabbits. If this incidence peak reflects an incidence peak in the same definitive rabbit hosts, then it supposes a second seasonal peak of tularemia in rabbits not previously appreciated. If *D. variabilis* and *A. americanum* are the primary vectors for maintenance of both of these peaks of incidence, all stages of *D. variabilis* and the nymphs of *A. americanum* would supposedly be vectors during the spring-early summer enzootic tularemia cycle based on their seasonal questing activity and host feeding preferences. Only larval *D. variabilis* and larval *A. americanum* would be expected to be involved in the late summer fall incidence peak because these larval stages exhibit some degree of questing activity during the late summer-fall incidence peak for feline tularemia.

Two other tick species possibly involved in maintenance of the supposed late summer-fall enzootic incidence peak are *Haemaphysalis leporispalustris* and *Ixodes scapularis*. The rabbit tick, *H. leporispalustris,* exhibits a feeding preference for rabbits and a bimodal season questing pattern with spring-summer and fall-winter peaks of questing activity and mid-summer and mid-winter nadirs. The larval stage of *H. leporispalustris,* which preferentially feeds on rabbits is active during the fall with peak questing activity in September and October [41, 42]. The fall feline tularemia which peaks in October (Fig. 1) can be attributed to sick rabbits exposed to *F. tularensis* vectored via larval *H. leporispalustris*. Parker et al. have observed the transstadial and transovarial transmission of *F. tularensis* in *H. leporispalustris* and have shown that these ticks to be naturally infected with *F. tularensis* [43]. A survey of tick infestations of cottontail rabbits in northwestern Alabama found *H. leporispalustris* (all life stages) to be the most abundant tick found on rabbits (79.8 % of all ticks collected over a 4-year period) [41]. Thus, the rabbit tick has the potential to be a major player in maintaining tularemia in the rabbit population.

Hopla observed in experiments reported in 1962 that *I. scapularis* larvae could be infected with *F. tularensis* by acquisition feeding, maintain infection through transstadial molts to the adult stage, and transmit the infection to laboratory animals. *I. scapularis* is an abundant tick species in the central US tularemia endemic region with all three stages exhibiting some degree of questing activity during the late summer-fall period [44]. One potential inconsistency with the supposition that *I. scapularis* might be a vector for maintenance of the enzootic cycle in rabbits in the late summer-fall period is that *I. scapularis* larvae have a feeding preference for skinks in Oklahoma, where both Hopla’s and Morton’s data was collected. Furthermore, *I. scapularis* nymphs and larvae are not very active during late summer-fall, and adults although very active during this period, have feeding preferences for larger mammals such as canids and cervids [45]. However, Hopla in his study did collect *I. scapularis* nymphs from a skink which were naturally infected with *F. tularensis* and after molting to adults in the laboratory transmitted the infection to a laboratory rabbit [44]. The potential role of reptiles as a definitive host for maintenance of enzootic tularemia in the central US endemic region has received little attention.

## Human Tularemia

Since the 1980s, human tularemia in the central USA occurs primarily as a tick transmitted incidental infection that does not contribute to maintenance of the tularemia endemic cycle [1]. Tick species implicated as bridging vectors from the enzootic definitive hosts to humans are *D. variabilis* and *A. americanum* [1, 2, 18, 19•]. Human tularemia incidence in Oklahoma during the same years as the data for feline tularemia demonstrated a late-spring summer incidence peak with a shoulder into late summer-early fall (Fig. 1, addition of data for human tularemia in Oklahoma for the years 2007 to 2014 show similar seasonality for human tularemia to the data included in Fig. 1). The late-spring summer peak is coincident with the questing activity for all stages of both *D. variabilis* and *A. americanum*. Feeding preferences for the various stages of these ticks, suggests that adult *D. variabilis* and nymph and adult *A. americanum* might be most active bridging vectors for human tularemia in the central US tularemia endemic region.

The spring-early summer enzootic rabbit tularemia peak, after accounting for the incubation period in cats, would slightly precede feline peak incidence. This delay between the enzootic rabbit tularemia peak and the incidence maxima in humans is compatible with our supposition that it takes a little more than a month for the ticks to acquire the infection from rabbits, molt and then transmit the disease to humans with a typical incubation period of 3 to 14 days for tick bite tularemia in humans.

The late summer-fall shoulder of the incidence in human tularemia in Oklahoma is more difficult to relate to its source. This shoulder may be attributed to direct transmission as well as questing activity for the proposed bridging vectors, particularly nymph and adult *A. americanum*, extending in some years into this season. The lack of a second seasonal incidence peak for human tularemia in the central US endemic region may be related to diminish or the absence of an active bridging vector during the fall-winter enzootic tularemia season.

## Conclusions

Domestic cats are highly susceptible to infection and acquire tularemia by predation of infected rabbits. Use of the seasonal pattern of feline tularemia as a surrogate for the seasonality of the rabbit enzootic cycle suggests that rabbit tularemia exhibits a bimodal seasonality with peaks in the spring and fall. The spring peak coincides with peak questing activity for two primary tick vectors, *D. variabilis* and *A. americanum*, whereas the fall peak does not correspond well with the questing activity of these ticks. It does, however, correspond with the questing activity of the rabbit tick, *H. leporispalustris,* which has peak questing activity in both the spring and fall. Human tularemia in this endemic region is primarily a tick transmitted disease with a late-spring, early summer peak incidence corresponding to the peak questing activity of its proposed primarily tick bridging vectors *D. variabilis* and *A. americanum*. However, while human tularemia has a late-summer fall shoulder of incidence, it does not exhibit a second peak of incidence like feline tularemia. This may be because either the questing activity of its bridging tick vectors is either diminished or absent during this season.

The study of the ecology of tularemia is complicated by the diverse array of animals and arthropod vectors *F. tularensis* can infect in nature. Recent evidence showing high mortality in eastern cottontail rabbits weakens the long held reservoir status of that species. Feline tularemia seasonal incidence may be a good reflection of the disease in the enzootic rabbit population. Further in-depth field and experimental studies will advance our knowledge of the ecology of tularemia in this endemic region.
